# Relationship Between Critical Power and Different Lactate Threshold Markers in Recreational Cyclists

**DOI:** 10.3389/fphys.2021.676484

**Published:** 2021-06-09

**Authors:** Pedro L. Valenzuela, Lidia B. Alejo, Almudena Montalvo-Pérez, Jaime Gil-Cabrera, Eduardo Talavera, Alejandro Lucia, David Barranco-Gil

**Affiliations:** ^1^Faculty of Sport Sciences, Universidad Europea de Madrid, Madrid, Spain; ^2^Physical Activity and Health Laboratory, Instituto de Investigación Sanitaria Hospital ‘12 de Octubre’ (‘imas12’), Madrid, Spain

**Keywords:** endurance performance, cycling, peak power output, Dmax method, incremental test

## Abstract

**Purpose:** To analyze the relationship between critical power (CP) and different lactate threshold (LT2) markers in cyclists.

**Methods:** Seventeen male recreational cyclists [33 ± 5 years, peak power output (PO) = 4.5 ± 0.7 W/kg] were included in the study. The PO associated with four different fixed (onset of blood lactate accumulation) and individualized (Dmax_exp_, Dmax_pol_, and LT_Δ1_) LT2 markers was determined during a maximal incremental cycling test, and CP was calculated from three trials of 1-, 5-, and 20-min duration. The relationship and agreement between each LT2 marker and CP were then analyzed.

**Results:** Strong correlations (*r* = 0.81–0.98 for all markers) and trivial-to-small non-significant differences (Hedges’ *g* = 0.01–0.17, bias = 1–9 W, and *p* > 0.05) were found between all LT2 markers and CP with the exception of Dmax_exp_, which showed the strongest correlation but was slightly higher than the CP (Hedges’ *g* = 0.43, bias = 20 W, and *p* < 0.001). Wide limits of agreement (LoA) were, however, found for all LT2 markers compared with CP (from ±22 W for Dmax_exp_ to ±52 W for Dmax_pol_), and unclear to most likely practically meaningful differences (PO differences between markers >1%, albeit <5%) were found between markers attending to magnitude-based inferences.

**Conclusion:** LT2 markers show a strong association and overall trivial-to-small differences with CP. Nevertheless, given the wide LoA and the likelihood of potentially meaningful differences between these endurance-related markers, caution should be employed when using them interchangeably.

## Introduction

The workload associated with the transition from steady to non-steady state oxidative metabolism (or heavy- to severe-intensity exercise) has been proposed as one of the main determinants of endurance performance (Poole et al., 2016). Knowledge of this workload might also be useful for training prescription given that training below this boundary level will induce different physiological responses and adaptations than intensities above it (Poole et al., 2016). However, numerous intensity markers or “thresholds” have been considered to describe this transition (Jones et al., 2019). For instance, the critical power (CP), the maximal lactate steady state (MLSS), or the respiratory compensation point (RCP) is among the most popular indicators of this threshold (Poole et al., 1988; Iannetta et al., 2020b).

The lactate threshold (LT2) determined during the incremental exercise in the laboratory setting is one of the most traditionally used markers for the assessment of endurance performance and the guidance of training intensity prescription (Joyner and Coyle, 2008; Faude et al., 2009; Iannetta et al., 2020b; Poole et al., 2021). The LT2 is typically defined as the maximum workload that precedes a rapid increase in blood lactate values resulting from an imbalance between lactate production and clearance, which is accompanied by a rise in blood H^+^ (Binder et al., 2008). It should be noted, however, that the workload at which blood lactate demonstrates an accelerated increase can be identified using a variety of methods (Faude et al., 2009). Moreover, while the lactate response to exercise might be reproducible under standardized conditions, physiological factors, such as muscle glycogen concentration or acid–base balance, and methodological factors, such as the testing protocol, can alter the workload at which the LT2 occurs (Jacobs, 1986; Faude et al., 2009; Jamnick et al., 2018).

Under this context, it has been proposed that CP should be considered the “gold standard” index of the transition between steady and non-steady state oxidative metabolism, being a stronger correlate of physical performance than the LT2 (Jones et al., 2019; Poole et al., 2021). Although different mathematical models exist, CP – usually determined through several constant-load tests to exhaustion or time trials of fixed duration – is based on the hyperbolic relationship between external workload [which in the sport of cycling is normally expressed as power output (PO)] and time to fatigue, in which CP (in watts) is the asymptote of this relationship and W' (in joules) is the curvature constant (Monod and Scherrer, 1965). As with the LT2, CP is also related to endurance performance (Kolbe et al., 1995; Smith et al., 1999), although the CP concept is unique with regard to physiological “thresholds” (e.g., LT2 and RCP) in that its definition is based purely on the measurement of mechanical work done (Jones et al., 2019).

CP and LT2 seem, therefore, to represent similar theoretical concepts, and both are widely used among researchers and coaches for training control and performance assessment. Nevertheless, controversy exists on whether LT2 markers can be used as surrogates of CP (McLellan and Cheung, 1992; Clingeleffer et al., 1994; Smith and Jones, 2001; Dekerle et al., 2003). Accordingly, the aim of the present study was to analyze the relationship between different LT2 markers and CP in recreational cyclists.

## Materials and Methods

### Participants

Seventeen male recreational cyclists (age = 33 ± 5 years, body mass = 72 ± 6 kg, and height = 178 ± 5 cm) participated in the study. Inclusion criteria included cycling a minimum of 4 h per week and a cycling experience greater than 2 years. Participants were instructed to maintain their normal dietary pattern for the duration of the study and to refrain from performing intense exercise and consuming ergogenic aids, such as caffeine 48 h prior to each testing session. In agreement with the Committee of Ethics of the University of Alcalá (CEI/HU/2017/26), all participants signed an informed consent form after having the procedures verbally explained.

### Experimental Design

Participants visited the laboratory on two different occasions interspersed by a minimum of 48 h, completing all procedures in a maximum of 1 week. All tests were performed on the same validated indoor cycle trainer (CycleOps, Madison, WI; Lillo-Bevia and Pallarés, 2018), allowing participants to use their own bicycle. During the first session, participants performed a maximal incremental test to characterize the blood lactate curve, which was then modeled using different approaches (Machado et al., 2011, 2012; Gavin et al., 2012; Santos-Concejero et al., 2014). In the second session, participants performed three-time trials of different duration for the computation of CP. All sessions included an initial warm-up consisting of light cycling (~100 W) for 10 min at a self-selected cadence.

### Lactate Threshold Determination

Participants performed a maximal incremental cycling test with an initial workload of 150 W, which increased by 25 W every 3 min until volitional exhaustion or when pedaling cadence was less than 60 rpm. This incremental protocol was designed attending to the recommendations by Bentley and co-workers (Bentley et al., 2007), who concluded that 3-min stages represent the optimal option for measuring both maximal and submaximal physiological values. The PPO was determined as the highest, fully completed stage during the incremental maximal test. If the last stage was not fully completed, PPO was calculated according to the formula proposed elsewhere (De Pauw et al., 2013).

**Figure 1:**
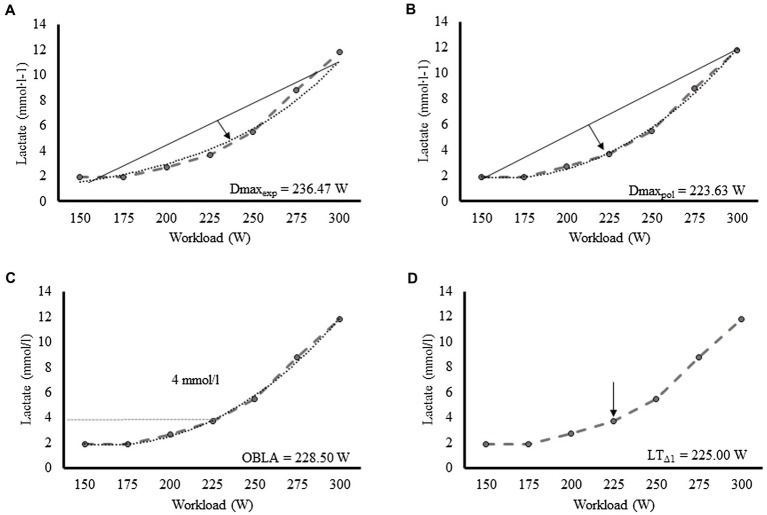
Representative lactate curve of a participant showing the determination of Dmax_exp_ **(A)**, Dmax_pol_ **(B)**, OBLA **(C)**, and LT_Δ1_ **(D)**. OBLA, onset of blood lactate accumulation.

Before and at the end of the incremental test as well as after each completed 3-min stage, 0.5 μl capillary blood samples were drawn from the ear lobe for lactate analysis (Lactate Scout, SensLab GmbH, Germany). The following four LT2 markers were determined with the Lactate-E software (Newell et al., 2007): (1) Dmaxesp (Figure 1A) was determined from the lactate-power data fitted by an exponential plus constant regression curve (Machado et al., 2011), corresponding to the point on the regression curve that yielded the maximal perpendicular distance to the straight line connecting the first and last point of the curve; (2) Dmax_pol_ (Figure 1B) was determined as described for Dmax_exp_, with the difference that a third-order polynomial regression curve was used to fit the lactate and power data instead of an exponential plus constant regression curve (Machado et al., 2012; Santos-Concejero et al., 2014); (3) The onset of blood lactate accumulation (OBLA; Figure 1C), which corresponded to the power equivalent to a blood lactate concentration of 4 mmol/l determined by interpolation from a third-order polynomial regression model of the lactate vs. work load curve; and (4) LT_Δ1_ (Figure 1D) considered as the point at which blood lactate values increased ≥1 mmol/l with regard to the previous stage (Gavin et al., 2012). These LT2 markers have been widely used in the scientific literature and proven to be related to endurance performance (Gavin et al., 2012; Machado et al., 2012; Santos-Concejero et al., 2014).

### Critical Power Determination

Participants performed three-time trials of 1, 5, and 20 min – always in this order – and the mean PO was registered. The three trials were performed in the same session and were interspersed by 30 min of passive recovery, as previously reported (Galbraith et al., 2014; Triska et al., 2015; Karsten et al., 2021). No specific pacing strategy was recommended, although participants were encouraged to achieve the highest mean PO possible during the trials. Participants were allowed to change gears during the test so as to maintain their preferred cadence.

Critical power was determined as the slope of the regression line of work against time following the equation (Moritani et al., 1981):

$$\mathrm{Work}\;\left(Jules\right)=W^{\prime }\left(Jules\right)+CP\;\left(Watts\right)\cdot \mathrm{Duration}\;\left(\sec\right)$$

### Statistical Analysis

Normality (Kolmogorov–Smirnov test) and homoscedasticity (Levene’s test) of the data were checked prior to any statistical treatment. Differences between each LT2 marker and CP were determined using Student’s paired *t*-tests. The magnitude of the differences (effect size) was assessed using Hedges’ *g* and were considered trivial (*g* < 0.20), small (*g* < 0.60), moderate (*g* < 1.20), large (*g* < 2.00), or very large (*g* > 2.00; Hopkins et al., 2009). The bias and limits of agreement (LoA) between markers were assessed using Bland–Altman plots. Differences between markers were also assessed through magnitude-based inferences with a 90% confidence level setting two different smallest worthwhile changes (SWC; Batterham and Hopkins, 2006; Karsten et al., 2021). An SWC of 1% was chosen because this value has been reported to represent a meaningful change in performance in cyclists (i.e., above the random error of measurement and also above the typical day-to-day variation in performance; Paton and Hopkins, 2001, 2006; Hopkins, 2004). An SWC of 5% was also analyzed given that variations in PO of this magnitude have been reported to suffice for modifying the steady physiological response to exercise at CP (Poole et al., 1988). Pearson’s correlation analysis and Lin’s concordant coefficient were used to examine the relationship between markers. Values of *r* of 0.1, 0.3, 0.5, 0.7, and 0.9 were considered small, moderate, large, very large, and extremely large, respectively (Hopkins et al., 2009). The standard error of estimate was used to examine the relationship between the different markers. Data are shown as mean ± SD. Statistical analyses were performed with a spreadsheet (Hopkins, 2015) and statistical software (SPSS 26.0, Inc., Chicago, IL), setting the alpha for significance at 0.05.

## Results

Participants’ PPO was 321 ± 40 W (or 4.5 ± 0.7 W/kg). The mean PO associated with the Dmax_exp_, Dmax_pol_, OBLA, and LT_Δ1_ was 273 ± 49 (84.6 ± 6.0% of PPO), 252 ± 42 (78.6 ± 9.4% of PPO), 261 ± 53 (80.7 ± 8.7% of PPO), and 247 ± 43 W (76.5 ± 7.2% of PPO), respectively. Participants’ mean PO during the 1-, 5-, and 20-min time trials was 477 ± 72, 318 ± 55, and 265 ± 45 W, respectively. The resultant CP was 252 ± 44 W (78.3 ± 6.2% of PPO).

All LT2 markers were strongly correlated with each other (Values of *r* ranging from 0.73 to 0.95), although small but significant differences were found between Dmax_exp_ and the other LT2 markers examined (bias ranging from 9 to 18 W), as well as between OBLA and LT_Δ1_ (bias = 11 W; Table 1).

**Table 1:** Relationship between the power output associated with different lactate threshold markers.

	Relationship			Agreement			
	*r*	*p*	SEE (W)	LoA (W)	CCC	Student’s *t*-test Value of *p*	Hedges’ *g*
Dmax_exp_−Dmax_pol_	0.73	<0.001	23.6	18.4 ± 33.5	0.659	0.020	0.45
Dmax_exp_−OBLA	0.95	<0.001	11.2	8.7 ± 15.8	0.926	0.008	0.22
Dmax_exp_−LT_Δ1_	0.84	<0.001	18.4	14.3 ± 26.0	0.717	<0.001	0.56
Dmax_pol_−OBLA	0.82	<0.001	21.2	16.5 ± 30.1	0.791	0.223	0.19
Dmax_pol_−LT_Δ1_	0.76	<0.001	21.0	16.4 ± 29.8	0.754	0.474	0.12
OBLA−LT_Δ1_	0.93	<0.001	13.9	10.8 ± 19.7	0.877	0.008	0.29

CCC, Lin’s concordant coefficient; OBLA, onset of blood lactate accumulation; LoA, limit of agreement presented as bias ± 1.96* standard deviation; and SEE, standard estimated error.

**Figure 2:**
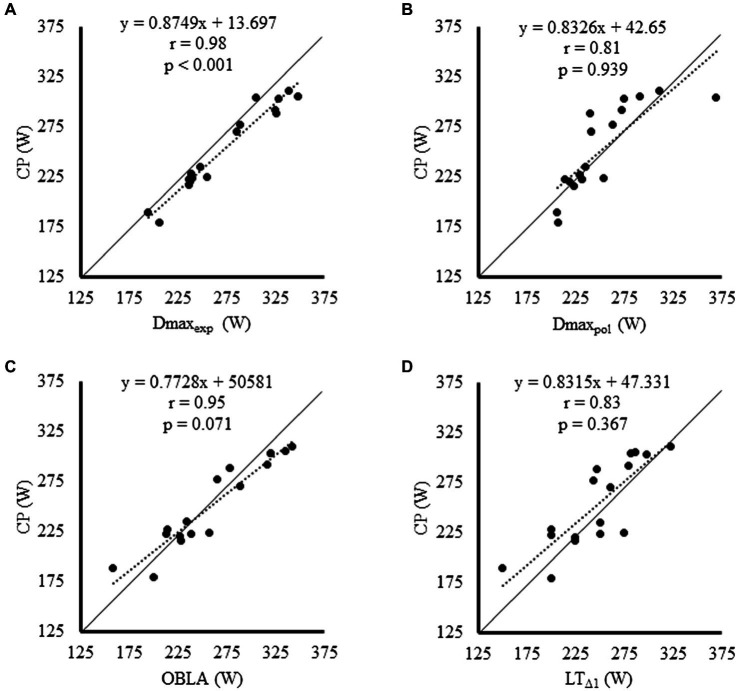
Relationship between each lactate threshold marker [Dmax_exp_ **(A)**, Dmax_pol_ **(B)**, OBLA **(C)**, and LT_Δ1_ **(D)**] and critical power. OBLA, onset of blood lactate accumulation.

**Figure 3:**
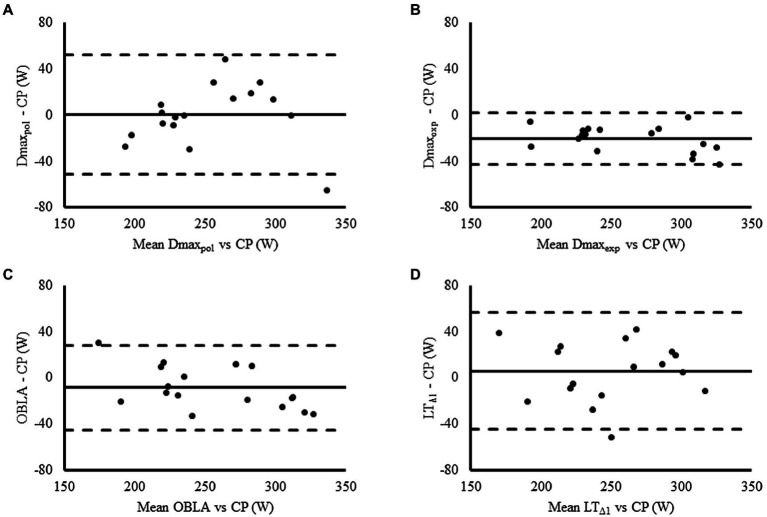
Bland–Altman plot displaying the agreement between each lactate threshold marker and critical power [Dmax_exp_ **(A)**, Dmax_pol_ **(B)**, OBLA **(C)**, and LT_Δ1_ **(D)**]. Solid and dashed horizontal lines represent the bias and the limits of agreement (bias ± 1.96 SD), respectively.

The relationship and agreement of each LT2 marker with CP are shown in Figures 2, 3, respectively. CP was significantly and strongly correlated with all LT2 markers (Values of *r* ranging from 0.81 for Dmax_pol_ to 0.98 for Dmax_exp_; Table 2). Non-significant and trivial-to-small differences were found between all LT2 markers and CP (bias ranging from 1 to 9 W) except for Dmax_exp,_ which was significantly and largely higher than CP (bias = 20 W; Table 2). Wide LoA were, however, found for all LT2 markers when compared with CP, which ranged between ±22 W for Dmax_exp_ and ±52W for Dmax_pol_ (Figure 3; Table 2). The likelihood of differences >1% between CP and LT2 markers was 100% (most likely), 33% (unclear), 82% (likely), and 70% (unclear) for Dmax_exp_, Dmax_pol_, OBLA, and LT_Δ1_, respectively, whereas the likelihood of differences >5% with CP was 2% (unclear), 0% (unclear), 0% (unclear), and 0% (unclear), respectively.

**Table 2:** Relationship and agreement between the power output corresponding to different lactate threshold markers and critical power.

		Relationship with CP				Agreement with CP			
	*r*	*p*	SEE (W)	LoA (W)	CCC	Student’s *t*-test Value of *p*	Hedges’ *g*
Dmax_pol_	0.81	<0.001	26.4	0.5 ± 51.9	0.810	0.939	0.01
Dmax_exp_	0.98	<0.001	9.9	−20.4 ± 22.3	0.878	<0.001	0.43
OBLA	0.95	<0.001	14.6	5.8 ± 50.4	0.912	0.071	0.17
LT_Δ1_	0.83	<0.001	25.5	−8.7 ± 36.5	0.817	0.367	0.13

CP, critical power; CCC, Lin’s concordant coefficient; LoA, limit of agreement presented as bias ± 1.96* standard deviation; OBLA, onset of blood lactate accumulation; and SEE, standard estimated error.

## Discussion

In the present study, we found that LT2 markers – including the fixed marker OBLA and the individualized markers Dmax_exp_, Dmax_pol_, or LT_Δ1_ – are strongly correlated with CP in recreational cyclists. Also, no significant differences were observed between the LT2 markers and CP, with the exception of Dmax_exp_. However, the wide LoA and the likelihood of practically meaningful differences (>1%, which would be of relevance for performance purposes) found between these indices raise concern on whether they could be used interchangeably.

Different markers associated with the so-called “anaerobic threshold” have been compared with CP in the scientific literature, and mixed results have been reported for their association (Galán-Rioja et al., 2020). In a recent meta-analysis, CP was reported to be significantly correlated with different indices, such as the first and second ventilatory threshold (the latter also known as “RCP”) or the MLSS, but the LT2 was not assessed (Galán-Rioja et al., 2020). Regarding the latter, CP (or “critical velocity” for running exercise) has been reported to be significantly correlated with the individual anaerobic threshold (McLellan and Cheung, 1992) and with the OBLA (Housh et al., 1991; Denadai et al., 2005), although controversy exists over its association with the LT2 determined by visual inspection (Smith and Jones, 2001). Our study suggests that most LT2 markers – at least those assessed here – are associated with CP, although the strength of this association might partially depend on the analyzed marker, with Dmax_exp_ emerging as the most strongly correlated (*r* = 0.98). Given that CP has been proposed as the gold standard index of the heavy- to severe-intensity exercise transition (Jones et al., 2019; Poole et al., 2021) and has proven to be strongly related to endurance performance (Kolbe et al., 1995; Smith et al., 1999), our findings support the assessment of LT2 markers and, particularly, Dmax_exp_ as an alternative method to estimate CP and predict endurance performance. Indeed, the previous studies have shown the superiority of Dmax_exp_ over other LT2 markers (including OBLA, Dmax_pol_, or LT_Δ1_) for the prediction of endurance performance (Nicholson and Sleivert, 2001; Machado et al., 2011, 2012; Santos-Concejero et al., 2014).

Despite their potential association, mixed evidence exists regarding the agreement of equivalence between LT2 markers and CP. For instance, CP (or critical speed) has been reported to be higher than the individual anaerobic threshold (McLellan and Cheung, 1992) and that the OBLA (Housh et al., 1991; Clingeleffer et al., 1994) in some studies, but others reported no significant differences between CP and the LT2 determined by visual inspection (Smith and Jones, 2001) or between CP and OBLA (determined as a blood lactate concentration of 3.5 mmol/l instead of 4.0 mmol/l; Denadai et al., 2005). Our results indicate that, besides being strongly correlated, CP and LT2 markers showed overall trivial-to-small differences. Nevertheless, despite the strong correlations and the absence of significant differences, wide LoA [from ±22 W (~10%) to ±52 W (~20%)] and some likelihood (33–100%) of differences that might be practically relevant for performance purposes (i.e., >1%, which is above the random error for the variability of cyclists’ performance between days) were found between markers. It must be noted, nonetheless, that the likelihood of differences between markers >5% was unclear. Differences >5% might have real-world relevance for training purposes. Although exercise at CP may not always elicit steady-state physiological responses (Overend et al., 1992; Brickley et al., 2002; Pringle and Jones, 2002; Sawyer et al., 2012; Mattioni Maturana et al., 2016), it is accepted that exercise below vs. above (~+5%) this threshold does differentiate between heavy and severe exercise on the basis of several physiological profiles (e.g., oxygen uptake, lactate, and pH; Poole et al., 1988; Jones et al., 2008). Similarly, there is disagreement on whether exercising at the LT2 elicits a steady physiological response, which might partly depend on the LT2 marker analyzed (Schnabel et al., 1982; Stegmann and Kindermann, 1982; Ribeiro et al., 1986; Oyono-Enguelle et al., 1990; Urhausen, 1993; Baldari and Guidetti, 2000; Lajoie et al., 2000; Jamnick et al., 2018) as well as on the protocol used for LT2 determination (Simon et al., 1983). Thus, the present findings suggest that LT2 markers are unlikely to overestimate or underestimate CP by more than 5%. However, given that small variations in exercise intensity (probably <5%) can result in different physiological responses and adaptations, caution should be taken when indistinctly using these markers for training prescription (Granata et al., 2018; Iannetta et al., 2020a).

Some methodological considerations of the present study deserve comment. We aimed to compare an “effort-dependent” marker obtained during several time trials, CP, with an “effort-independent” marker obtained during incremental exercise, the LT2. Effort-independent markers of physiologic thresholds have been reported to have some benefits over effort-independent markers because they do not rely on participants’ volition or perceptions, being preferable for testing vulnerable populations (e.g., patients or very old people; Poole et al., 2021). However, the LT2 determination can be affected by several variables including the specific marker analyzed, glycogen store status, acid–base balance, or testing protocol (e.g., stage duration; Jacobs, 1986; Faude et al., 2009; Jamnick et al., 2018; Poole et al., 2021). For this reason, it has been proposed that when individuals are able to undertake high-intensity exhausting exercise, CP offers the greatest potential to predict athletic performance, assess physiological function, and monitor training efficacy (Poole et al., 2021). Moreover, as previously reported for gas exchange thresholds (e.g., RCP), the comparison of physiological markers, such as LT2 obtained during incremental exercise with others assessed during constant exercise (i.e., CP), might induce some bias due to an overestimation of the former (Iannetta et al., 2019; Caen et al., 2021), although an incremental test with 3-min stages, such as the one used in the present study, is expected to be sufficient to elicit a steady physiological state (Bentley et al., 2007). On the other hand, different LT2 markers have been used in the scientific literature (Faude et al., 2009), but here, we just analyzed some of the most popular ones and therefore, different LT2 markers might yield different results. The protocol used for the determination of CP can also affect its magnitude (Triska et al., 2018). In this regard, following the protocol used in the previous studies (Galbraith et al., 2014; Triska et al., 2015; Karsten et al., 2021), we conducted a single-day protocol for the assessment of CP with a 30-min rest between time trials, which has been reported to provide similar CP estimates compared with other protocols with a longer rest period (Galbraith et al., 2014). However, it might be hypothesized that fatigue could potentially impair the PO attained during the time trials and consequently the estimation of CP. We are unable to ascertain whether this was actually the case in the present study as no physiological verifications of the CP estimates were performed. Therefore, our findings should be confirmed using different protocols.

In summary, the present study indicates a strong association between different LT2 markers and CP in recreational cyclists. However, although differences between LT2 markers and CP were overall trivial-to-small, the wide LoA found and the likelihood of practically meaningful differences (albeit lower than 5%) raise concerns on the suitability of using these markers interchangeably for training prescription, as they might potentially induce different physiological responses and adaptations.

## Data Availability Statement

The original contributions presented in the study are included in the article/supplementary material, and further inquiries can be directed to the corresponding author.

## Ethics Statement

The studies involving human participants were reviewed and approved by the Committee of Ethics of the University of Alcalá (CEI/HU/2017/26). The patients/participants provided their written informed consent to participate in this study.

## Author Contributions

Conception and design of the experiments and drafting of the manuscript: PV. Experimental preparation and data collection: PV, DB-G, LA, AM-P, JG-C, and ET. Analysis and interpretation: PV, DB-G, LA, AM-P, JG-C, ET, and AL. Revision of the manuscript for important intellectual content: All authors. All authors contributed to the article and approved the submitted version.

### Conflict of Interest

The authors declare that the research was conducted in the absence of any commercial or financial relationships that could be construed as a potential conflict of interest.
